# Endothelial Nitric Oxide Synthase Single Nucleotide Polymorphism and Left Ventricular Function in Early Chronic Kidney Disease

**DOI:** 10.1371/journal.pone.0116160

**Published:** 2015-01-22

**Authors:** Sourabh Chand, Colin D. Chue, Nicola C. Edwards, James Hodson, Matthew J. Simmonds, Alexander Hamilton, Stephen C. L. Gough, Lorraine Harper, Rick P. Steeds, Jonathan N. Townend, Charles J. Ferro, Richard Borrows

**Affiliations:** 1 Department of Nephrology, Queen Elizabeth Hospital Birmingham, Birmingham, B15 2WB, United Kingdom; 2 Centre for Translational Inflammation Research, University of Birmingham, Birmingham, B15 2WB, United Kingdom; 3 Department of Cardiology, Queen Elizabeth Hospital Birmingham, Birmingham, B15 2WB, United Kingdom; 4 Department of Statistics, Wolfson Laboratory, Old Queen Elizabeth Hospital, Birmingham, B15 2TH, United Kingdom; 5 Oxford Centre for Diabetes, Endocrinology and Metabolism, University of Oxford, Churchill Hospital, Headington, Oxford, OX3 7LJ, United Kingdom; 6 Oxford National Institute for Health Research Biomedical Research Centre, Churchill Hospital, Oxford, OX3 7LG, United Kingdom; University Medical Center Utrecht, NETHERLANDS

## Abstract

**Background:**

Chronic kidney disease (CKD) is associated with accelerated cardiovascular disease and heart failure. Endothelial nitric oxide synthase *(eNOS)* Glu298Asp single nucleotide polymorphism (SNP) genotype has been associated with a worse phenotype amongst patients with established heart failure and in patients with progression of their renal disease. The association of a cardiac functional difference in non-dialysis CKD patients with no known previous heart failure, and *eNOS* gene variant is investigated.

**Methods:**

140 non-dialysis CKD patients, who had cardiac magnetic resonance (CMR) imaging and tissue doppler echocardiography as part of two clinical trials, were genotyped for *eNOS* Glu298Asp SNP retrospectively.

**Results:**

The median estimated glomerular filtration rate (eGFR) was 50mls/min and left ventricular ejection fraction (LVEF) was 74% with no overt diastolic dysfunction in this cohort. There were significant differences in LVEF across eNOS genotypes with GG genotype being associated with a worse LVEF compared to other genotypes (LVEF: GG 71%, TG 76%, TT 73%, p = 0.006). After multivariate analysis, (adjusting for age, eGFR, baseline mean arterial pressure, contemporary CMR heart rate, total cholesterol, high sensitive C-reactive protein, body mass index and gender) GG genotype was associated with a worse LVEF, and increased LV end-diastolic and systolic index (p = 0.004, 0.049 and 0.009 respectively).

**Conclusions:**

*eNOS* Glu298Asp rs1799983 polymorphism in CKD patients is associated with relevant sub-clinical cardiac remodelling as detected by CMR. This gene variant may therefore represent an important genetic biomarker, and possibly highlight pathways for intervention, in these patients who are at particular risk of worsening cardiac disease as their renal dysfunction progresses.

## Introduction

Chronic kidney disease (CKD) is a major public health issue, mainly due to accelerated cardiovascular disease, affecting an estimated 10–16% of the population in developed countries[1, 2]. Non-traditional risk factors and early cardiovascular changes in CKD have been increasingly recognised to lead to heart failure and sudden cardiac death related cardiovascular mortality, implicating left ventricular disease [3, 4]. The determinants of the severity of myocardial disease are poorly characterised though hypertension, oxidative stress and activation of the renal angiotensin system are all thought to be relevant. Research into the genetic predisposition to the development of heart failure in CKD has been limited [5].

In the general population, there has been interest in the association between the Glu298Asp polymorphism within endothelial nitric oxide synthase (*eNOS*) and heart failure [6, 7]. Although this polymorphism has been associated with endothelial dysfunction and progression of CKD through nitric oxide effects [8], it is not known if this polymorphism is associated with early cardiac structural changes that occur in non-dialysis CKD. In light of this, we investigated if this gene variant is associated with changes in systolic and diastolic function, based on detailed cardiac magnetic resonance imaging (CMR) in non-dialysis CKD patients with no known history of heart failure.

## Methods

### Patients

The study cohort consisted of patients who were initially enrolled into two completed, single centre, randomised controlled trials (CRIB-II [9] and CRIB-PHOS [10]) based at the Queen Elizabeth Hospital Birmingham. To limit the confounding effect of population stratification only white patients (self-reported ethnicity) were included in this genetic substudy (n = 140). Inclusion criteria were: baseline CMR and echocardiographic investigations, age 18–80 with non-dialysis CKD, total cholesterol <5.5mmol/L, resting blood pressure controlled to <140/90 for at least 6months. Exclusion criteria were: diabetes mellitus, peripheral vascular disease, previous myocardial infarction, known heart failure, valvular heart disease and atrial fibrillation. The study was approved by East Midlands Nottingham 1 Research Ethics Committee and adhered to the Declaration of Helsinki. Study participants provided written informed consent.

### Genotyping

Whole blood (8.5ml) was collected in PAXgene Blood DNA Tubes (Qiagen, Manchester, UK) which were then frozen and stored at -80°C. Samples were defrosted at room temperature for 2 hours before starting the DNA extraction process. DNA extraction was undertaken using the PAXgene Blood DNA Kit (Qiagen Manchester, UK) as previously described [11]. All DNA samples were then all diluted to a working stock of 4ng/µl with 2.25µl of DNA added into each reaction. *eNOS* (G894T) SNP rs1799983 genotyping was performed using Taqman technology as previously described [12]. 384-well plates were read using a 7900HT Fast Real-Time PCR System (Applied Biosystems, Foster City, USA).

### Cardiovascular Magnetic Resonance Imaging

CMR was performed on a 1.5-T scanner (Sonata Symphony, Siemens, Erlangen, Germany). Serial contiguous short axis cines were piloted from the vertical long axis and horizontal long axis of the right and left ventricle (electrocardiogram gated, steady-state free precession imaging [True-Fisp]; temporal resolution 40–50ms, repetition time 3.2ms, echo time 1.6ms, flip angle 60°, slice thickness 7mm) in accordance with previously validated methodologies [13]. Analysis was performed off-line (Argus Software, Siemens) by two blinded observers (NE and CDC) for the assessment of ventricular volumes (end-diastole, end-systole, stoke volume) and ejection fraction. Heart rate and baseline brachial blood pressure was measured at the time of CMR.

### Echocardiography

Transthoracic echocardiography (Vivid 7; GE Vingmed Ultrasound, Horten, Norway) was performed by an experienced echocardiographer. All parameters were measured in triplicate according to American Society of Echocardiography recommendations [14] and analysed offline (EchoPAC; GE Vingmed Ultrasound, Horten, Norway) by two blinded observers (NE and CDC ). Resting left ventricular (LV) diastolic function was assessed using standard techniques [15].

### Arterial Stiffness and Distensibility

Pulse wave velocity, augmentation index and ascending aortic distensibility were common measures of arterial stiffness and distensibility in the two randomised control trials, they have been included here. Pulse wave analysis was performed on the radial artery using a high-fidelity micromanometer (SPC-301; Miller Instruments, Houston, TX). The peripheral arterial waveform was used to generate a central arterial waveform using a validated transfer function (SphygmoCor; AtCor Medical, Sydney, Australia). The same system was used to determine aortic pulse wave velocity by sequentially recording ECG-gated carotid and femoral waveforms as previously described [16]. CMR data was used for the ascending aortic distensibility measurement data.

### Outcome Measures

The primary aim was to evaluate the association between LV ejection fraction (LVEF) with *eNOS* genotype. Secondary outcomes included: LV end-diastolic volume indexed to body surface area (LVEDVI), LV end-systolic volume indexed to body surface area (LVESVI) and S’ lat (systolic velocity of the basal anterolateral LV wall). As there is much debate over which parameters of diastolic function should be measured as well as their variability in assessment [17], six diastolic parameters were assessed in a cluster analysis (see statistical analysis below) to separate the cohort into two risk categories of diastolic dysfunction; these were then assessed by *eNOS* genotype. The “diastolic” parameters included: e’ lat (early diastolic velocity of the basal anterolateral LV wall on tissue doppler), E/e’ lat (maximum velocity of the E-wave of mitral valve inflow by the maximal anterolateral LV wall velocity of e’), mitral valve E/A (ratio of early to late mitral inflow velocities), LVMI (left ventricular mass index), mitral valve propagation velocity (MV VP) and LAVI (left atrial volume index).

### Statistical Analysis

Baseline demographics and cardiac investigations were compared across the genotype groups, using Kruskal-Wallis (continuous data) and Fisher’s Exact tests (categorical data) as appropriate. The relationship between these was then investigated using regression analysis. Initially, univariate linear regression models were produced, with variables being log-transformed where there was evidence of a non-linearity. Multivariate regression models were used (including all factors simultaneously) in order to adjust for potentially confounding factors.

A cluster analysis was then performed, to divide patients into groups based on the values of a range of diastolic parameters. The Two-Step cluster analysis in IBM SPSS 19 was used, with the number of clusters determined automatically (in this study, two main clusters). Demographic factors were then compared between the resulting clusters, using t-tests or Fisher’s Exact test, as applicable. All analyses were performed using IBM SPSS 19 (IBM Corp. Armonk, NY), with p<0.05 deemed to be indicative of statistical significance.

## Results

Genomic DNA was successfully genotyped in 132 (>94%) patients. The *eNOS* SNP rs1799983 patient genotype frequency was GG in 47% (62/132), TG in 39% (51/132), and TT in 14% (19/132). This distribution was within Hardy-Weinberg equilibrium bounds (p>0.05). Patient demographics are presented in Table 1 for the cohort as a whole and stratified by genotype (GG, TG and TT).There were no significant demographic differences across the three genotypes. Median age was 57 years, eGFR was 50.5 mls/min/1.73m^2^, with 85% of patients prescribed an angiotensin converting enzyme inhibitor or angiotensin II receptor blocker. The most common renal disease group was glomerular disease, with 16% of the cohort patients been diagnosed with IgA nephropathy on renal histology.

**Table 1:** Baseline Demographics (p value across the three genotype groups).

**Characteristic**	**GG genotype**	**TG genotype**	**TT genotype**	**All**	**p value**
Number of patients (%)	62 (47)	51 (39)	19 (14)	132	
Age (years)	57 (46–63)	59 (47–66)	58 (42–69)	57 (46–65)	0.690
Estimated glomerular filtration rate (mls/min/1.73m^2^)	50 (40–61)	51 (38–56)	45 (32–59)	51 (38–59)	0.686
Male (%)	30 (48)	28 (55)	15 (79)	73 (55)	0.063
High sensitive C-reactive protein (mg/l)	1.59 (0.57–5.00)	1.75 (0.91–5.97)	2.79 (0.67–9.87)	1.87 (0.72–5.62)	0.550
Total cholesterol (mmol/l)	4.7 (4.4–5.4)	4.5 (4.0–5.0)	4.8 (3.6–5.9)	4.6 (4.0–5.2)	0.374
Mean arterial pressure (mmHg)	92 (85–101)	90 (85–100)	91 (85–99)	91 (85–100)	0.898
Systolic blood pressure (mmHg)	129 (117–139)	127 (114–135)	124 (111–139)	127 (115–139)	0.539
Diastolic Blood Pressure (mmHg)	72 (66–81)	73 (67–81)	73 (66–79)	73 (66–80)	0.938
Body surface area (m^2^)	1.87 (1.77–2.02)	1.90 (1.73–2.03)	1.97 (1.92–2.13)	1.92 (1.79–2.03)	0.061
Body mass index (Kg/m^2^)	27.5 (24.1–31.4)	27.5 (24.2–31.1)	29.2 (25.7–32.2)	27.7 (24.2–31.4)	0.350
Brain natriuretic peptide (ng/L)	86.6 (34.9–176.2)	84.3 (30.9–205.7)	70.0 (37.1–160.9)	84.4 (33.8–193.0)	0.989
Current Smoker (%)	8 (13)	5 (10)	2 (11)	15 (11)	0.930
Previous Smoker (%)	24 (39)	13 (25)	8 (42)	45 (34)	0.250
*Diagnoses*					
Glomerular diseases (%)	25 (40)	18 (35)	11 (58)	54 (40)	0.230
Systemic Diseases (%)	10 (16)	11 (22)	1 (5)	22 (17)	0.296
Tubulointerstitial diseases (%)	7 (11)	9 (18)	0	16 (12)	0.124
Familial nephropathies (%)	9 (15)	7 (14)	1 (5)	17 (13)	0.681
Miscellaneous (%)	11 (18)	6 (12)	6 (32)	23 (17)	0.147
*Medication frequency*					
Angiotensin conversion enzyme inhibitors (%)	35 (56)	29 (57)	14 (74)	78 (60)	0.401
Angiotensin II receptor blockers (%)	20 (32)	15 (29)	4 (21)	39 (30)	0.681
Β blockers (%)	13 (21)	10 (20)	3 (16)	26 (20)	0.956
Calcium channel blockers (%)	13 (21)	16 (31)	5 (26)	34 (26)	0.449
Alpha blockers (%)	7 (11)	6 (12)	2 (11)	15 (11)	0.999
Diuretics (%)	14 (23)	18 (35)	5 (26)	37 (28)	0.311
Statins (%)	28 (45)	20 (39)	9 (47)	57 (43)	0.765

Univariate analysis revealed a significant difference across genotypes for LVEF and LVESVI (p = 0.006 and p = 0.024 respectively, Table 2). Post hoc Mann-Whitney U testing demonstrated significant differences between GG genotype and both TG and TT genotypes (p<0.05 for both). Because of this, and to align with existing literature, further analysis compared the GG group with the non-GG (TG+TT) group, i.e. a “dominant” model. Linear regression analysis revealed an absolute 4% lower LVEF (p = 0.005) and a 21% relative increased LV end-systolic volume index (p = 0.011) in GG versus non-GG (TG+TT) genotyped patients as shown in Table 3. In multivariate analysis, (adjusting for age, gender, estimated glomerular filtration rate (eGFR), CMR heart rate (HR), total cholesterol, high sensitive C-reactive protein (hsCRP), body mass index (BMI—if variables were indexed accounting for body surface area, BMI was excluded in the analysis) and brachial mean arterial pressure (BMAP)) the GG genotype and male sex were associated with significantly lower LVEF (p = 0.004 and p = 0.017 respectively; Table 3). Male gender, GG genotype, eGFR, and heart rate were independently associated with a higher end systolic and diastolic volume index. A trend for GG genotype association with lower S^’^ lat was seen, but this failed to reach statistical significance (p = 0.106). As shown in S1 Table, increased aortic stiffness was associated age, male gender and BMAP but not GG genotype; ascending aortic distensibility decreased with age.

**Table 2:** Cardiac investigations relationship to genotype (p value across the three genotype groups).

**Cardiac Investigations**	**GG genotype**	**TG genotype**	**TT genotype**	**All**	**p value**
*Cardiac Magnetic Resonance Imaging*					
Left ventricular ejection fraction (%)	71 (65–76)	76 (71–80)	73 (66–78)	74 (68–77)	**0.006**
Left ventricular end-diastolic volume index (mls/m^2^)	61 (52–70)	57 (51–66)	59 (51–64)	59 (52–66)	0.344
Left ventricular end-systolic volume index (mls/m^2^)	17 (13–23)	14 (12–17)	16 (12–20)	16 (12–21)	**0.024**
Left Ventricular Mass Index (g/m^2^)	54.0 (45.0–67.0)	54.6 (45.0–67.1)	56.0 (47.0–61.9)	54.1 (45.3–65.7)	0.996
Ascending Aortic Distensibility (x10–^3^ mmHg^−1^)	2.53 (1.24–4.19)	2.15 (1.18–3.31)	2.11 (1.08–3.41)	2.35 (1.24–3.68)	0.692
CMR Cardiac Output (l/min)	5.12 (4.37–6.17)	5.19 (4.64–5.94)	6.38 (4.37–7.57)	5.26 (4.57–6.30)	0.177
CMR Heart Rate (beats/min)	66 (60–77)	64 (58–77)	78 (62–87)	66 (60–78)	0.074
*Tissue Doppler Echocardiography*					
S’ lat, cm/s	8 (7–10)	9 (7–10)	9 (7–11)	8 (7–10)	0.688
e’ lat, cm/s	9 (7–12)	9 (8–12)	9 (8–11)	9 (8–12)	0.790
E/e’ lat	7.17 (5.73–8.96)	6.95 (6.08–8.40)	6.73 (6.13–7.80)	7.00 (6.00–8.56)	0.786
Mitral valve inflow E/A ratio	1.02 (0.87–1.23)	0.95 (0.81–1.20)	0.93 (0.70–1.12)	0.97 (0.81–1.20)	0.273
Mitral valve propagation velocity	50.3 (41.9–66.7)	50.5 (43.6–65.1)	45 (37.7–65.8)	50.0 (41.0–66.4)	0.701
Left Atrial Volume Index (mls/m^2^)	26.3 (21.8–32.9)	24.5 (20.6–32.3)	27.3 (22.5–30.5)	26.2 (21.6–32.3)	0.760
Pulse wave velocity (m/s)	8.3 (7.3–9.5)	8.3 (7.2–10.0)	9.5 (6.6–10.7)	8.3 (7.2–10.0)	0.808
Augmentation Index (%)	29.0 (22.8–35.1)	27.3 (20.0–35.3)	27.2 (13.3–33.8)	28.3 (20.7–35.0)	0.691

Key: CMR (cardiac magnetic resonance); S’ lat (systolic velocity of the basal anterolateral LV wall); e’ lat (early diastolic velocity of the basal anterolateral LV wall on tissue doppler); E/e’ lat (maximum velocity of the E-wave of mitral valve inflow by the maximal anterolateral LV wall velocity of e’)

**Table 3:** Univariate and multivariate analysis of systolic function as compared to GG vs non-GG.

**Factor**	**Univariate**		**Multivariate**	
	***Coefficient (95% CI)***	***Sig.***	***Coefficient (95% CI)***	***Sig.***
***LVEF***				
Age	0.07 (−0.04, 0.18)	0.228	0.07 (−0.05, 0.19)	0.274
eGFR	0.00 (−0.09, 0.08)	0.915	0.03 (−0.06, 0.11)	0.541
BMAP	−0.09 (−0.21, 0.03)	0.158	−0.06 (−0.19, 0.06)	0.318
CMR HR	0.02 (−0.09, 0.12)	0.737	0.00 (−0.12, 0.12)	0.987
Total Cholesterol	−0.68 (−2.14, 0.77)	0.354	−0.70 (−2.32, 0.92)	0.394
Log_2_ hsCRP^‡^	0.03 (−0.75, 0.81)	0.936	−0.22 (−1.02, 0.58)	0.586
BMI	0.18 (−0.11, 0.47)	0.218	0.13 (−0.18, 0.45)	0.392
Gender (Female)	3.10 (0.30, 5.90)	**0.030**	3.62 (0.66, 6.58)	**0.017**
GG (Yes)	−3.95 (−6.70, −1.21)	**0.005**	−4.24 (−7.12, −1.35)	**0.004**
***LVEDVI***				
Age	−0.01 (−0.20, 0.17)	0.878	0.03 (−0.16, 0.21)	0.782
eGFR	0.16 (0.03, 0.30)	**0.015**	0.14 (0.01, 0.27)	**0.033**
BMAP	−0.14 (−0.34, 0.06)	0.169	−0.18 (−0.38, 0.01)	0.066
CMR HR	−0.20 (−0.37, −0.04)	**0.018**	−0.23 (−0.41, −0.04)	**0.017**
Total Cholesterol	−0.83 (−3.15, 1.49)	0.478	0.65 (−1.84, 3.15)	0.606
Log_2_ hsCRP^‡^	−1.09 (−2.32, 0.15)	0.084	−1.20 (−2.40, 0.00)	0.050
Gender (Female)	−4.68 (−9.17, −0.18)	**0.041**	−5.02 (−9.56, −0.48)	**0.031**
GG (Yes)	4.04 (−0.45, 8.53)	**0.078**	4.46 (0.02, 8.90)	**0.049**
***LVESVI*** ^#^				
Age	−0.40% (−1.00%, 0.20%)	0.195	−0.3% (−0.9%, 0.3%)	0.271
eGFR	0.50% (0.10%, 0.90%)	**0.021**	0.4% (0.0%, 0.8%)	0.076
BMAP	−0.20% (−0.80%, 0.50%)	0.614	−0.3% (−0.9%, 0.3%)	0.336
CMR HR	−0.53% (−1.07%, 0.02%)	0.059	−0.6% (−1.2%, 0.0%)	0.057
Total Cholesterol	0.3% (−7.0%, 8.2%)	0.936	3.5% (−4.7%, 12.3%)	0.413
Log_2_ hsCRP^‡^	−1.9% (−5.8%, 2.2%)	0.357	−1.4% (−5.3%, 2.6%)	0.474
Gender (Female)	−16.6% (−28.0%, −3.5%)	**0.015**	−19.4% (−30.6%, −6.4%)	**0.005**
GG (Yes)	21.0% (4.6%, 40.0%)	**0.011**	21.9% (5.3%, 41.1%)	**0.009**
***S’ Lat***				
Age	−0.03 (−0.07, 0.00)	**0.040**	−0.04 (−0.08, 0.00)	**0.040**
eGFR	0.00 (−0.03, 0.02)	0.759	−0.01 (−0.03, 0.02)	0.606
BMAP	0.02 (−0.02, 0.06)	0.294	0.02 (−0.02, 0.06)	0.248
CMR HR	−0.01 (−0.04, 0.02)	0.576	−0.02 (−0.06, 0.02)	0.347
Total Cholesterol	0.13 (−0.28, 0.55)	0.529	0.15 (−0.36, 0.65)	0.571
Log_2_ hsCRP^‡^	0.00 (−0.22, 0.22)	0.988	0.00 (−0.24, 0.25)	0.992
BMI	0.03 (−0.06, 0.11)	0.543	0.05 (−0.04, 0.15)	0.278
Gender (Female)	0.30 (−0.51, 1.11)	0.465	0.39 (−0.53, 1.30)	0.405
GG (Yes)	−0.45 (−1.26, 0.36)	0.270	−0.73 (−1.62, 0.16)	0.106

p-Values from linear regression analysis

#Outcome was log_2_-transformed prior to analysis to normalise the distribution. Quoted coefficients represent the percentage increase in the outcome for an increase in one of the factors (or for the stated category relative to the reference).

‡hsCRP was log_2_-transformed, hence the quoted coefficients relate to an increase of one unit in the log (i.e. a two-fold increase)

Key: eGFR (estimated glomerular filtration rate; BMAP (brachial mean arterial pressure); CMR HR (cardiac magnetic resonance heart rate); hsCRP (high sensitive C-reactive protein; BMI (body mass index)

A cluster analysis was performed based on diastolic parameters, in order to group patients based on their risk of diastolic function (Table 4). The most important factor in defining the clusters was found to be e’ lat, with mitral valve E/A (MV E/A) and E/e’ lat being moderate contributors, and the remaining parameters being minimally discriminative. The first cluster had lower levels of e’ lat, MV E/A and MV VP and higher levels of E/e’ lat and LAVI, hence represented those patients at most risk of diastolic dysfunction. Comparisons between the clusters found that increased age (p<0.001) and reduced eGFR (p = 0.014) were significantly associated with the risk of diastolic dysfunction clusters on univariate analysis (Table 5).

**Table 4:** Relative importance of diastolic parameters and their respective values for cluster separation.

**Factor**	**Relative Importance**	**Cluster 1**	**Cluster 2**
e’ lat	1.00	7.8 (7.4–8.3)	12.7 (11.9–13.5)
Mitral valve inflow E/A ratio	0.73	0.87 (0.82–0.92)	1.27 (1.18–1.37)
E/e’ lat	0.62	8.1 (7.6–8.6)	5.7 (5.4–6.1)
Mitral valve velocity propagation	0.37	46.5 (43.3–49.9)	67.6 (58.8–77.7)
Left Atrial Volume Index	0.12	27.0 (25.4–28.6)	23.8 (22.4–25.4)
Left Ventricular Mass Index	0.00	54.5 (51.1–58.1)	54.1 (50.3–58.2)

Data reported as: “Mean (95% CI)”.

Key: e’ lat (early diastolic velocity of the basal anterolateral LV wall on tissue doppler); mitral valve E/A (ratio of early to late mitral inflow velocities); E/e’ lat (maximum velocity of the E-wave of mitral valve inflow by the maximal anterolateral LV wall velocity of e’)

**Table 5:** Univariate analysis of diastolic parameters clusters.

**Factor**	**Cluster 1**	**Cluster 2**	**p-Value**
Age	60.9 (1.1)	43.8 (1.7)	**<0.001**
Estimated Glomerular Filtration Rate	47.3 (1.8)	54.5 (2.3)	**0.014**
Brachial Mean Arterial Pressure	92.2 (1.4)	91.4 (1.9)	0.700
*Gender (Male)*	37 (56.1%)	19 (48.7%)	0.545
*Genotype (GG)*	28 (42.4%)	22 (56.4%)	0.225

Continuous factors are reported as: “Mean (SE)”, with p-values from independent sample t-tests.

Dichotomous factors are reported as: “N (%)”, with p-values from Fisher’s Exact Test.

## Discussion

In this cohort of white patients with non-dialysis dependent CKD, and without heart failure, GG genotype for *eNOS* SNP rs1799983 was associated with a significant lower LVEF, greater LVESVI and greater LVEDVI than those found in non-GG genotypes. The burden of myocardial disease in CKD suggests the investigation of stratification by genetic risk in this setting to be a worthwhile endeavour, and this study represents the first such attempt with this *eNOS* polymorphism.

Previous data from the general population suggest this gene variant represents an attractive candidate SNP, and support the findings of the current study. For instance Velloso et al studied a multi-ethnic Brazilian population and demonstrated increased frequency of GG genotype in patients with systolic heart failure compared with healthy controls [7]. Another Brazilian study showed GG genotype was associated with a near 5% reduction in LVEF compared with TT genotype patients, findings very similar to those of the current study [18]. Also noteworthy is the higher all-cause mortality associated with the GG genotype in hypertensive patients [19]. An important aspect of the current study is the inclusion of white patients only, in an attempt to reduce confounding by population stratification. Indeed this is highlighted by the study of Velloso et al which did indeed show differences in genotype frequency at this locus between White and Afro-Brazilian individuals [7]. It should be acknowledged, however, that further validation of these findings in diverse populations are required to confirm the robustness of our (and other’s) findings.

The functional change associated with this gene variant also supports the clinical data. This polymorphism results from the nucleotide guanine substituting thiamine at position 894 of exon 7 on chromosome 7, and results in different cleavage of the eNOS enzyme depending on genotype [20]. The GG genotype of the studied SNP is associated with increased eNOS activity and nitric oxide levels [21, 22] and experimental overexpression of eNOS (which is present within ventricular myocytes) results in reduced ventricular function [21, 23]. This is particularly the case in conditions of oxidative stress such as CKD[24], since “uncoupling” of eNOS may lead to generation of superoxide anion radicals that further exacerbate cardiac dysfunction [25].

The influence of genotype on cardiac function and outcome may be context-specific. Of note, McNamara et al suggested a beneficial effect of GG genotype outcome in patients with established, clinically evident heart failure [6]. Whilst at first sight this data conflicts with the current study, and with that of other reports [7, 8], it should be noted that 84% of patients displayed an ejection fraction ≤35% (97.2% of participants were in NYHA stage II or greater, with >50% in stages III or IV). Furthermore there were differences in age and aetiology (ischaemic versus non-ischaemic) between genotype groups which may have influenced the results as well as variation in the technique used in measuring ejection fraction. Thus, it is certainly possible that this *eNOS* SNP influences outcome differentially depending on the stage of heart failure studied. Although the present study’s exclusion criteria (diabetes mellitus, peripheral vascular disease, myocardial infarction, known heart failure, valvular heart disease, atrial fibrillation, dialysis-dependence and uncontrolled hypertension) limits the generalizability of its findings, the exclusion criteria does allow removal of these potential external factors that affect both eNOS activity and left ventricular function, allowing a more ‘pure’ analysis of *eNOS* polymorphism association with LVEF in early CKD. Long-term follow-up of the present study population is also desirable to monitor how these patients’ LVEFs and heart failure symptoms develop as their CKD progresses, in relation to their *eNOS* genotype.

This study benefits from a uniform technique of detailed CMR assessment of cardiac volumes and systolic function, and very careful clinical phenotyping. Although no association with “diastolic dysfunction” parameters derived from echocardiography and genotype was evident, the size of the cohort means that such an effect cannot be excluded, and further study in larger cohorts is required.

In summary, *eNOS* Glu298Asp polymorphism in non-dialysis CKD patients is associated with relevant sub-clinical cardiac remodelling as detected by CMR. This gene variant may therefore represent an important genetic biomarker, and possibly highlight pathways for intervention, in these patients who are at particular risk of worsening cardiac disease as their renal dysfunction progresses.

## Supporting Information

S1 Table Univariate and multivariate analysis of arterial stiffness and arterial distensibility as compared to GG vs non-GG. (DOCX)
