# Supplementary material for: Endothelial Nitric Oxide Synthase Single Nucleotide Polymorphism and Left Ventricular Function in Early Chronic Kidney Disease
Source: PLoS One. 2015 Jan 22;10(1):e0116160. doi: 10.1371/journal.pone.0116160 (PMC4303420; doi:10.1371/journal.pone.0116160)
Supplement: S1 Table — (DOCX) [file pone.0116160.s001.docx]

| **Factor** | **Univariate** | | **Multivariate** | |
| --- | --- | --- | --- | --- |
|  | ***Coefficient (95% CI)*** | ***Sig.*** | ***Coefficient (95% CI)*** | ***Sig.*** |
| ***Pulse Wave Velocity^#^*** | | | | |
| GG (Yes) | -1.1% (-9.8%, 8.5%) | 0.815 | -1.1% (-7.3%, 5.4%) | 0.725 |
| Age | 1.3% (1.0%, 1.6%) | **<0.001** | 1.3% (1.0%, 1.6%) | **<0.001** |
| Gender (Female) | 0.5% (-8.4%, 10.3%) | 0.913 | 7.2% (0.3%, 14.4%) | **0.039** |
| eGFR | -0.2% (-0.5%, 0.0%) | 0.095 | 0.0% (-0.2%, 0.1%) | 0.622 |
| BMAP | 0.9% (0.5%, 1.3%) | **<0.001** | 0.9% (0.6%, 1.2%) | **<0.001** |
| Total Cholesterol | 3.8% (-1.0%, 8.8%) | 0.122 | 2.6% (-1.0%, 6.4%) | 0.155 |
| Log_2_CRP^‡^ | 2.5% (-0.1%, 5.1%) | 0.057 | 0.7% (-1.1%, 2.5%) | 0.447 |
| BMI | 1.6% (0.7%, 2.5%) | **<0.001** | 0.7% (0.0%, 1.4%) | 0.060 |
| CMR HR | 0.2% (-0.1%, 0.6%) | 0.227 | 0.2% (0.0%, 0.5%) | 0.077 |
| ***Augmentation Index*** | | | | |
| GG (Yes) | 0.91 (-2.59, 4.40) | 0.609 | 0.14 (-3.02, 3.30) | 0.928 |
| Age | 0.27 (0.14, 0.41) | **<0.001** | 0.41 (0.28, 0.54) | **<0.001** |
| Gender (Female) | 6.13 (2.79, 9.48) | **<0.001** | 9.40 (6.16, 12.64) | **<0.001** |
| eGFR | 0.00 (-0.10, 0.11) | 0.936 | 0.03 (-0.07, 0.12) | 0.555 |
| BMAP | 0.19 (0.04, 0.34) | **0.013** | 0.22 (0.08, 0.35) | **0.002** |
| Total Cholesterol | -0.61 (-2.41, 1.20) | 0.508 | -0.31 (-2.08, 1.46) | 0.725 |
| Log_2_CRP^‡^ | 0.01 (-0.97, 0.99) | 0.982 | -0.13 (-1.00, 0.74) | 0.774 |
| BMI | 0.00 (-0.36, 0.36) | 0.988 | -0.18 (-0.52, 0.16) | 0.303 |
| CMR HR | -0.11 (-0.25, 0.02) | 0.104 | -0.10 (-0.23, 0.04) | 0.165 |
| ***Ascending Aortic Distensibility^#^*** | | | | |
| GG (Yes) | 1.9% (-24.7%, 38.0%) | 0.900 | -7.4% (-26.7%, 17.0%) | 0.515 |
| Age | -4.5% (-5.3%, -3.6%) | **<0.001** | -4.8% (-5.7%, -3.9%) | **<0.001** |
| Gender (Female) | 15.8% (-14.4%, 56.6%) | 0.339 | -10.3% (-29.2%, 13.5%) | 0.360 |
| eGFR | 0.4% (-0.5%, 1.3%) | 0.414 | 0.0% (-0.7%, 0.6%) | 0.914 |
| BMAP | -0.8% (-2.1%, 0.6%) | 0.246 | -0.9% (-1.9%, 0.1%) | 0.087 |
| Total Cholesterol | 4.7% (-10.3%, 22.2%) | 0.556 | 2.5% (-10.1%, 16.9%) | 0.708 |
| Log_2_CRP^‡^ | -1.6% (-9.4%, 6.9%) | 0.698 | -0.2% (-6.3%, 6.2%) | 0.939 |
| BMI | -0.4% (-3.5%, 2.7%) | 0.778 | 2.3% (-0.2%, 4.9%) | 0.069 |
| CMR HR | -0.1% (-1.2%, 1.1%) | 0.886 | -0.8% (-1.8%, 0.1%) | 0.093 |
| ***Left Ventricular Mass Index^#^*** | | | | |
| GG (Yes) | -1.6% (-10.2%, 7.8%) | 0.733 | -0.2% (-7.2%, 7.4%) | 0.965 |
| Age | 0.2% (-0.2%, 0.5%) | 0.348 | 0.0% (-0.3%, 0.3%) | 0.895 |
| Gender (Female) | -25.3% (-30.7%, -19.4%) | **<0.001** | -24.1% (-29.6%, -18.2%) | **<0.001** |
| eGFR | 0.3% (0.0%, 0.5%) | 0.050 | 0.3% (0.1%, 0.5%) | **0.008** |
| BMAP | 0.5% (0.1%, 0.9%) | **0.012** | 0.5% (0.1%, 0.8%) | **0.005** |
| Total Cholesterol | -2.9% (-7.4%, 1.7%) | 0.210 | -1.2% (-5.1%, 2.9%) | 0.555 |
| Log_2_CRP^‡^ | 0.4% (-2.1%, 2.8%) | 0.772 | 0.4% (-1.6%, 2.4%) | 0.724 |
| CMR HR | -0.5% (-0.8%, -0.2%) | **0.002** | -0.4% (-0.7%, 0.0%) | **0.026** |

p-Values from linear regression analysis

#Outcome was log_2_-transformed prior to analysis to normalise the distribution. Quoted coefficients represent the percentage increase in the outcome for an increase in one of the factors (or for the stated category relative to the reference).

‡hsCRP was log_2_-transformed, hence the quoted coefficients relate to an increase of one unit in the log (i.e. a two-fold increase)

Key: eGFR (estimated glomerular filtration rate; BMAP (brachial mean arterial pressure); CMR HR (cardiac magnetic resonance heart rate); hsCRP (high sensitive C-reactive protein; BMI (body mass index)
